# TIRAP, an Adaptor Protein for TLR2/4, Transduces a Signal from RAGE Phosphorylated upon Ligand Binding

**DOI:** 10.1371/journal.pone.0023132

**Published:** 2011-08-01

**Authors:** Masakiyo Sakaguchi, Hitoshi Murata, Ken-ichi Yamamoto, Tomoyuki Ono, Yoshihiko Sakaguchi, Akira Motoyama, Toshihiko Hibino, Ken Kataoka, Nam-ho Huh

**Affiliations:** 1 Department of Cell Biology, Okayama University Graduate School of Medicine, Dentistry and Pharmaceutical Sciences, Kita-ku, Okayama, Japan; 2 Interdisciplinary Research Organization, University of Miyazaki, Kiyotakecho, Miyazaki, Japan; 3 Shiseido Research Center, Kanazawa-ku, Yokohama, Japan; Roswell Park Cancer Institute, United States of America

## Abstract

The receptor for advanced glycation end products (RAGE) is thought to be involved in the pathogenesis of a broad range of inflammatory, degenerative and hyperproliferative diseases. It binds to diverse ligands and activates multiple intracellular signaling pathways. Despite these pivotal functions, molecular events just downstream of ligand-activated RAGE have been surprisingly unknown. Here we show that the cytoplasmic domain of RAGE is phosphorylated at Ser391 by PKCζ upon binding of ligands. TIRAP and MyD88, which are known to be adaptor proteins for Toll-like receptor-2 and -4 (TLR2/4), bound to the phosphorylated RAGE and transduced a signal to downstream molecules. Blocking of the function of TIRAP and MyD88 largely abrogated intracellular signaling from ligand-activated RAGE. Our findings indicate that functional interaction between RAGE and TLRs coordinately regulates inflammation, immune response and other cellular functions.

## Introduction

The receptor for advanced glycation end products (RAGE) is a type I single-pass transmembrane protein belonging to the immunoglobulin superfamily [1]. RAGE is thought to be involved in a broad range of inflammatory, degenerative and hyperproliferative diseases, including sepsis, rheumatoid arthritis, diabetic nephropathy, atherosclerosis, cancer, and neurological disorders [2]–[5]. The receptor is composed of an extracellular region (one V-type and two C-type immunoglobulin domains), a hydrophobic transmembrane-spanning domain, and a short cytoplasmic tail. The extracellular domain can bind diverse ligands, including advanced glycation end products (AGEs) [2], high-mobility group box 1 (HMGB1) [6], S100 family proteins [7], and amyloid fibrils [8].

Binding of ligands to RAGE leads to activation of multiple signaling pathways, including signaling pathways involved in the production of reactive oxygen species, Ras-mediated extracellular signal-regulated kinase 1/2 (ERK1/2), Akt, stress-activated protein kinase/c-Jun-NH2-terminal kinase (SAPK/JNK), p38 MAP kinase, small GTPase Cdc42/Rac1, and NFκB and AP-1 transcription factors [9]–. In accordance with this, cellular biological outcomes are also diverse and include induction of apoptosis, hyperproliferation, increased motility, and production of inflammatory cytokines [4], [5], [14]. It is intriguing that different RAGE ligands often show different effects in a given type of cell. For example, we previously showed that dimerized S100A11 activated Akt and induced production of EGF, thus stimulating growth of normal human keratinocytes via RAGE [15]. In the same cells, S100A8/A9, which is known to be a RAGE ligand [16], enhanced production of proinflammatory cytokines including IL-8, IL-1F9 and TNFα but neither induced EGF nor activated Akt [17].

RAGE has a short cytoplasmic domain (41 amino acids), from which RAGE-triggered signaling is initiated. The cytoplasmic domain has no obvious signaling domain or any motifs known for receptor signaling. Hudson et al. identified Diaphanous-1 (Dia-1) to be a binding partner to the RAGE cytoplasmic domain by employing a yeast two-hybrid method [18]. Dia-1 is required for controlling the activation of Rac-1 and Cdc42, resulting in promotion of cellular migration. It is unclear, however, whether diverse downstream signaling pathways of RAGE are controlled by Dia-1 alone. Ishihara et al. [19] reported that extracellular signal-regulated protein kinase-1 and -2 (ERK1/2) bind to the cytoplamic domain of RAGE, but the biological significance of the interaction was not clarified. It remains a major challenge to reveal how the cytoplasmic domain of RAGE is activated upon ligand binding and how its activation is transduced to downstream signaling molecules for biological significance of RAGE in normal and pathological circumstances.

In the present study, we focused on molecular events in and at the cytoplasmic domain of RAGE. We found that the cytoplasmic domain of RAGE was phosphorylated at Ser391 by PKCζ upon binding of ligands. To our surprise, TIRAP and MyD88, which are known to be adaptor proteins for TLR2/4, bound to the phosphorylated RAGE and transduced a signal to downstream molecules. The findings indicate that functional interaction between RAGE and TLRs coordinately regulates inflammation, immune response and other cellular functions.

## Results

### Cytoplasmic Domain of RAGE Is Phosphorylated by PKCζ upon Ligand Binding

**Figure 1:**
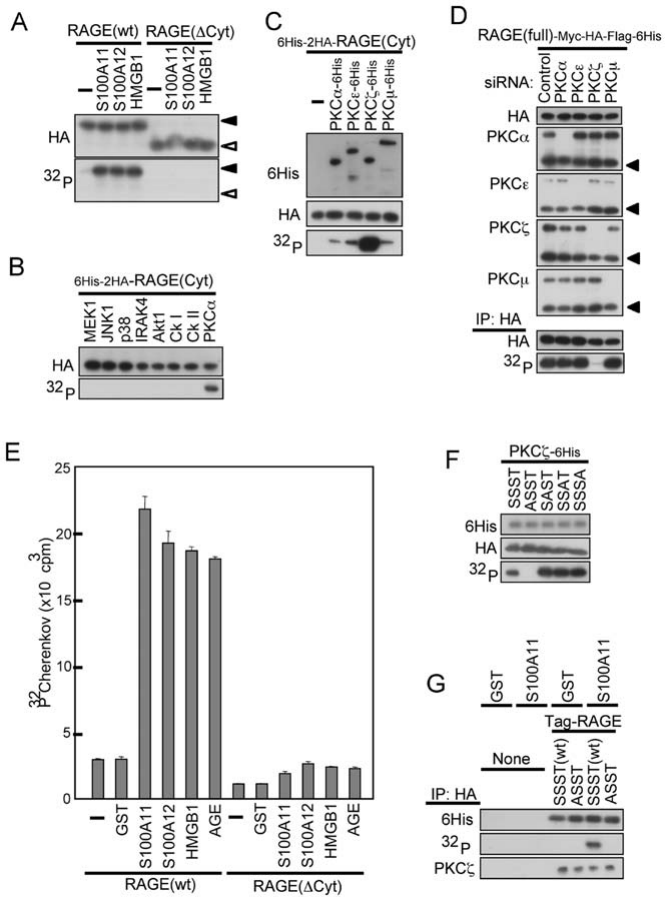
RAGE is phosphorylated at Ser391 by PKCζ upon ligand binding. (A) Phosphorylation of full-length RAGE (wt, closed arrowhead) but not cytoplasmic domain-deleted RAGE (ΔCyt: open arrowhead). HEK293 cells were transfected with epitope-tagged RAGE constructs (RAGE-Myc-HA-Flag-6His), [^32^P]phosphate-labeled, and treated with the ligands (10 ng/ml, 1 h), and then RAGE proteins were pulled down with anti-HA antibody (clone HA-7)-agarose before Western blotting and autoradiography. The results were confirmed by five similar experiments. (B and C) Phosphorylation of RAGE(Cyt) *in vitro*. 6His-2HA-RAGE(Cyt) purified from transfected HEK293 cells by anti-HA antibody (clone HA-7)-agarose was incubated with constitutively active recombinant kinases of commercial source. Western blotting with anti-HA and anti-6His antibodies was performed for determination of amounts of RAGE(Cyt) and kinase proteins, respectively. The results were confirmed by five similar experiments (B) or by an independent and four related experiments (C). (D) Down-regulation of endogenous PKCζ but not of other isoforms by siRNA abrogated S100A11 (10 ng/ml, 6 h)-induced phosphorylation of co-transfected RAGE-Myc-HA-Flag-6His in HEK293 cells. The results are representative of three independent experiments. (E) Activation of endogenous PKCζ by RAGE ligands. HEK293 cells transfected with RAGE-Myc-HA-Flag-6His (wt or ΔCyt) were treated with 10 ng/ml of GST, S100A11, S100A12, and HMGB1 or 100 µg/ml of AGE for 1 h. Cell extracts were incubated with a specific PKCζ-substrate in the presence of [γ-^32^P]-ATP. The experiment was performed in quadruplicate, and the results were confirmed by three related experiments. (F) Determination of phosphorylation site of RAGE(Cyt). Recombinant PKCζ-6His (top panel) was incubated with isolated variants of 6His-2HA-RAGE(Cyt) [SSST (wild-type), ASST (Ser391 to Ala), SAST (Ser399 to Ala), SSAT (Ser400 to Ala), SSSA (Thr401 to Ala)] and assayed as for (B) and (C). The results were confirmed by an independent and two related experiments. (G) S100A11 (10 ng/ml)-dependent phosphorylation of transfected 6His-2HA-RAGE(wt) at Ser391 in HEK293 cells. [^32^P]Phosphate-labeled cell extracts were pulled down with anti-HA agarose and immunoblotted with anti-6His antibody (top) or analyzed by autoradiography (middle). Co-precipitated endogenous PKCζ was detected with anti-PKCζ antibody (bottom). The results were confirmed by an independent and five related experiments.

We first examined whether the cytoplasmic domain of RAGE is phosphorylated. Overexpression of full-length RAGE but not that of a cytoplasmic domain-deleted construct resulted in phosphorylation in HEK293 cells, to which known RAGE ligands including S100A11, S100A12 and HMGB1 were applied (Figure 1A). Extent of the phosphorylation was S100A11 dose- and time-dependent (Figures S1A and S1B). Phosphorylation of RAGE was observed also upon stimulation of another RAGE ligands, S100A8/A9 (Figure S1C).

Since the cytoplasmic domain of RAGE lacks any potential kinase domain, we screened for a protein kinase(s) that can phosphorylate recombinant RAGE *in vitro* and identified PKCα among representative kinases examined (Figures 1B and S1D). Screening of PKC isotypes resulted in identification of PKCζ to be the most probable candidate for phosphorylation of RAGE (Figure 1C). Down regulation of endogenous PKCζ but not that of PKCα, PKCε and PKCµ abrogated S100A11-induced phosphorylation of RAGE in ^32^P-orthophosphate-labeled HEK 293 cells (Figure 1D). The specific siRNA against PKCζ also effectively inhibited S100A8/A9-induced phosphorylation of RAGE (Figure S1H). Endogenous PKCζ was activated by RAGE ligands such as S100A11, S100A12, HMGB1 and AGE in RAGE-transfected HEK293 cells as demonstrated by an *in vitro* assay showing phosphorylation of a PKCζ-specific substrate (Figure S1E) by the cell extracts (Figure 1E). Activity of PKCζ was dependent on dose of the ligands (Figure S1F). Extracts prepared from HEK293 cells transfected with cytoplasmic domain-deleted, hence signal transduction-deficient, RAGE did not show such an activity (Figure 1E).

The cytoplasmic domain of human RAGE has 4 potential phosphorylation sites, i.e., Ser391, Ser399, Ser400, and Thr401. Among the 4 potential phosphorylation sites of the cytoplasmic domain of RAGE, only Ser391 is conserved among humans, mice, Guinea pigs, rats, rabbits, dogs, and cats. Replacement of Ser391 with Ala, but not any of the other 3 residues, resulted in abrogation of phosphorylation by PKCζ *in vitro* (Figure 1F). Overexpressed wild-type RAGE but not the Ser391-replaced variant was phosphorylated in HEK293 cells when S100A11 was applied, while binding of PKCζ to RAGE was independent of the phosphorylation status (Figure 1G and S1G). These results indicated that RAGE is phosphorylated at Ser391 by PKCζ upon ligand binding.

### TIRAP binds to cytoplasmic domain of RAGE and transduces a signal from ligand-activated RAGE

The nature and functional mode of possible adaptor proteins for RAGE are not well understood. Our trial screening for proteins that bind to the cytoplasmic domain of RAGE by immunoprecipitation followed by mass spectrometry failed in identification of any promising candidates. We changed the strategy to a candidate-based screening partly because of implicated functional similarity between RAGE and TLRs [20]. Use of a potent expression vector and optimization of tags to express the short cytoplasmic domain of RAGE finally led to the identification of TIRAP as an adaptor protein for RAGE (Figure S2A).

**Figure 2:**
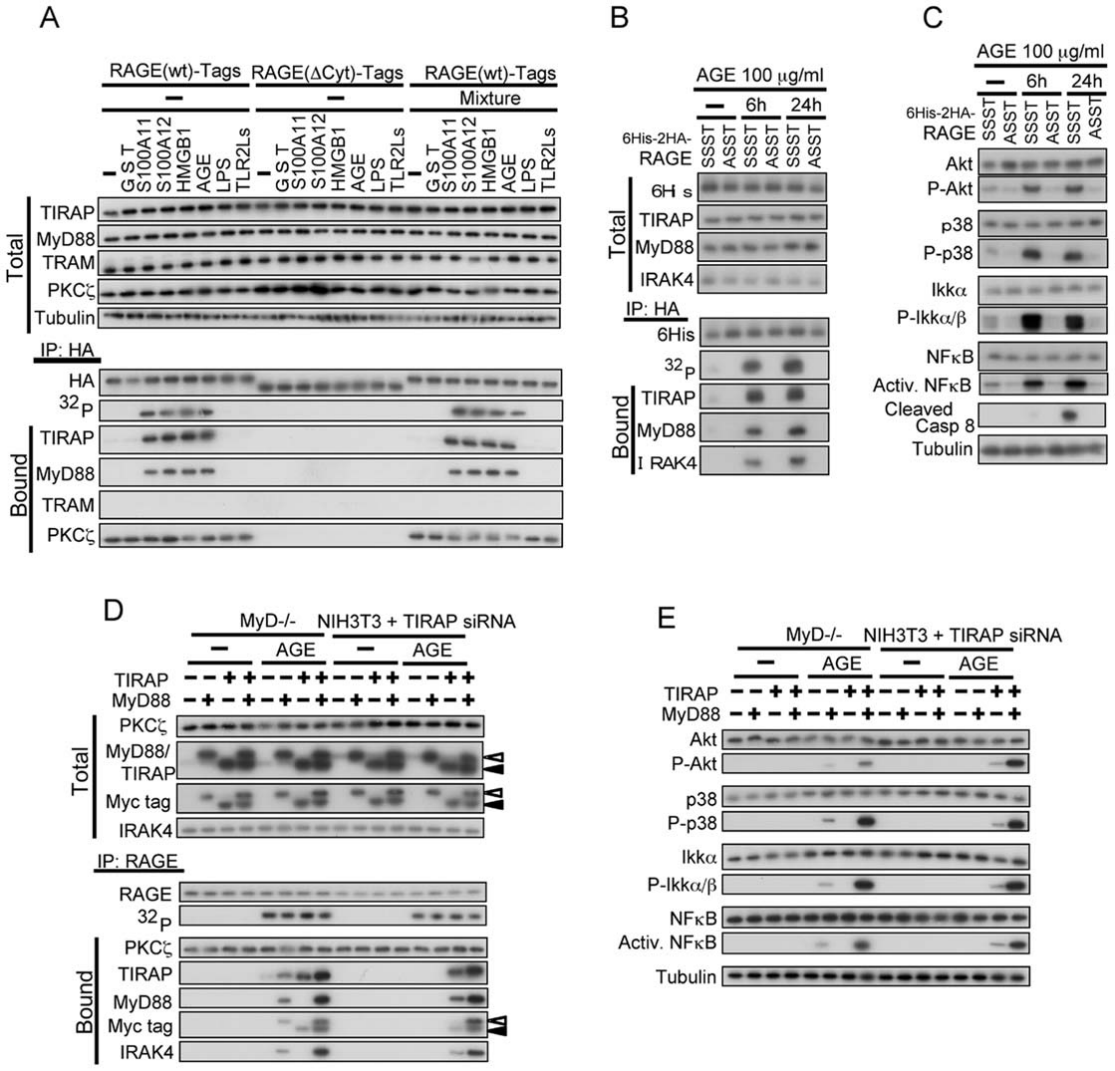
TIRAP and MyD88 function as adaptor proteins for RAGE. (A) Co-precipitation of endogenous TIRAP and MyD88 but not TRAM with recombinant RAGE(wt). HEK293 cells were transfected with RAGE(wt) and RAGE(ΔCyt) tagged with -Myc-HA-Flag-6His, [^32^P]phosphate-labeled, and treated with AGE at 100 µg/ml or with other ligands at 10 ng/ml for 1 h. TLR2/4 blocking mixture (Mixture, see Materials and Methods) was preincubated with the ligands for 30 min at room temperature. Cell extracts were analyzed by Western blotting with or without prior immunoprecipitation and by autoradiography. The results were confirmed by 7 related experiments. (B and C) AGE (100 µg/ml)-induced phosphorylation of RAGE at Ser391 is essential for recruitment of endogenous TIRAP and MyD88 (B: The results were confirmed by an independent and two related experiments.) and downstream signal transduction (C: The results were confirmed by an independent and two related experiments.) in HEK293 cells. Full-length 6His-2HA-RAGE constructs were used. SSST, wild-type; ASST, Ser391 to Ala. (D and E) Abortive signal transduction from activated endogenous RAGE in MyD88-/- or TIRAP-down regulated mouse fibroblasts was rescued by forced expression of the corresponding human genes. Mouse TIRAP siRNA and expression vectors were transfected 48 h before treatment with AGE (100 µg/ml, 1 h). Experiments were performed under the conditions similar to those described in (A) except for immunoprecipitation using a biotinylated antibody against endogenous RAGE. ^32^P shows the results of autoradiography of the immunoprecipitated RAGE. The results were confirmed by an independent and two related experiments.

TIRAP and MyD88, but not TRAM, were co-precipitated with overexpressed RAGE and RAGE was phosphorylated in HEK293 cells predominantly when the cells were treated with RAGE ligands, S100A11, S100A12, HMGB1 and AGE but not with a TLR4 ligand (LPS) and TLR2 ligands (Figure 2A). TLR2/4 blocker mixture did not affect ligand-induced activation of RAGE and its downstream signaling. The amount of TIRAP and MyD88 co-precipitated with the cytoplasmic domain of RAGE was shown to depend on phosphorylation status of Ser391 as demonstrated using phosphorylation-mimic and non-phosphorylatable variants (Figure S2B). In HEK293 cells, endogenous TIRAP, MyD88, and IRAK4 were co-precipitated with overexpressed wild-type RAGE but not with a non-phosphorylatable variant when the transfected cells were treated with AGE (Figure 2B). In accordance with this, downstream signal tansducers of TIRAP including Akt, p38, IKKα, NFκB, and caspase 8 were activated, as demonstrated by elevated phosphorylation levels for Akt, p38 and IKKα, increase in the amount of the truncated form for caspase 8, and increase in binding to NFκB-responsible element for NFκB (Figure 2C). Mutation in a protein-protein interaction domain of TIRAP resulted in loss of not only its binding to RAGE but also that of wild-type MyD88 (Figure S2C), indicating that MyD88 binds to RAGE only in the presence of TIRAP. Binding of TIRAP to the cytoplasmic domain of RAGE was confirmed to be direct using both recombinant proteins (data not shown). This mode of interaction is similar to that known for TLR2/4, TIRAP, and MyD88 [21]. Essential nature of TIRAP and MyD88 for RAGE signaling was demonstrated using TIRAP-down regulated NIH3T3 cells and MyD88-/- mouse fibroblasts (Figures S2D, 2D and 2E). Overexpression of TIRAP or MyD88 rescued the defective protein interaction and signal transduction in corresponding cells. These results indicate that TIRAP and MyD88 function as essential adaptor proteins for RAGE, binding to ligand-activated phosphorylated RAGE and transducing a signal from it.

### Signal transduction from activated endogenous RAGE and its biological consequence in human umbilical vein endothelial cells (HUVECs)

**Figure 3:**
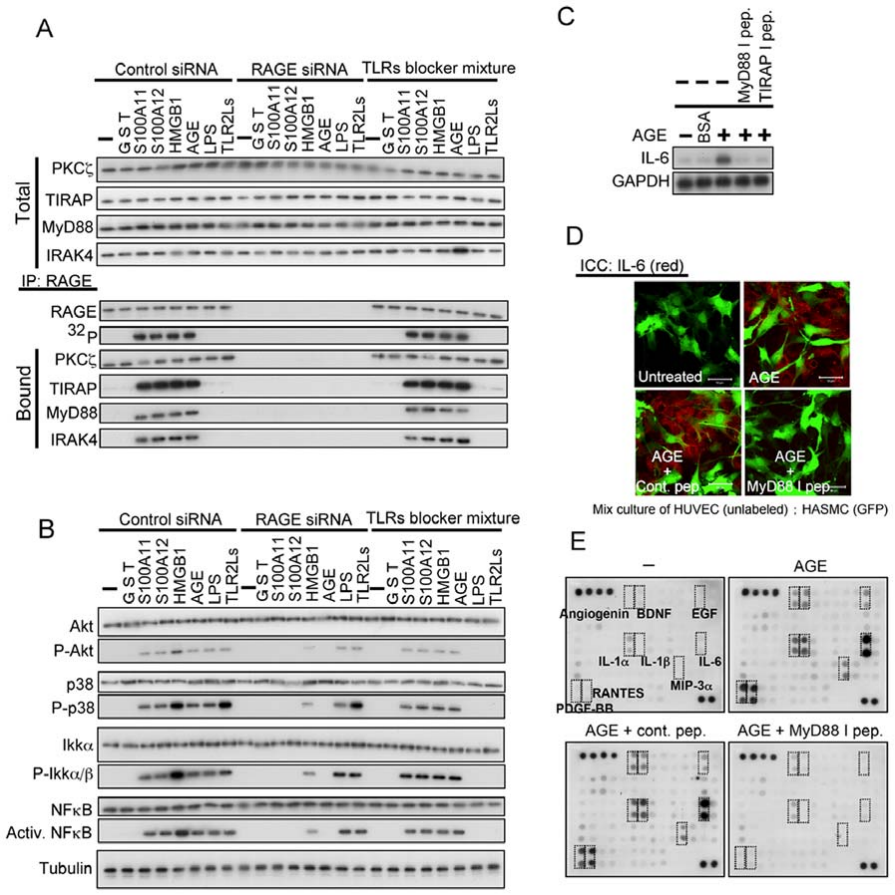
Specific activation of endogenous RAGE by AGE induced cytokines in HUVECs. (A and B) Activation of downstream signaling molecules by specific activation of endogenous RAGE. Experiments were performed under the conditions similar to those described in Fig. 2A except for down regulation of RAGE with siRNA, immunoprecipitation using an antibody against RAGE, and type of cells used. The results were confirmed by an independent and two related experiments (A) or by an independent and three related experiments (B). (C) Induction of IL-6 by AGE (100 µg/ml for 12 h) demonstrated by Northern blot analysis in HUVECs in serum- and supplement-free conditions. Cell-permeable inhibitory peptides (100 µM) were added 12 h prior to the addition of AGE. The results were confirmed by two independent and one related experiments. (D) Induction of IL-6 in HUVECs but not in HASMCs by treatment with AGE. HUVECs co-cultured with HASMCs pre-infected with an adenovirus carrying GFP were treated under conditions similar to those described in (C). Fixed cells were immunostained with anti-IL-6 antibody. Bars, 20 µm. The results were confirmed by three independent experiments. (E) Detection of cytokines secreted into the medium by HUVECs treated with AGE using an antibody array [Human cytokine Antibody Array VI & 6.1 (60), RayBiotech, Norcross GA]. Dotted squares indicate antibodies against corresponding cytokines. Cells were treated under conditions similar to those described in (C). The results were confirmed by an independent experiment.

We next examined activation and downstream signaling of endogenous RAGE using HUVECs. RAGE was expressed in HUVECs (Figure S3A). When HUVECs were exposed to AGE, the downstream signaling molecules from RAGE were activated in dose- and time-dependent manners (Figures S3B and S3C). Nuclear translocation of NFκB induced by AGE was abrogated by treatment with inhibitor peptides for MyD88 and TIRAP (Figure S3D). Application to HUVECs with RAGE ligands such as S100A11, S100A12, HMGB1, and AGE but not LPS and TLR2 ligands induced phosphorylation of endogenous RAGE, interaction with TIRAP, MyD88, and IRAK4, and transduction of signal to the downstream molecules (Figures 3A and 3B). A mixture of TLR2/4 blockers showed no effect on these processes. Activation of the molecules downstream from RAGE was abrogated by treatment of HUVECs with inhibitor peptides for MyD88 and TIRAP and siRNA for PKCζ (Figures S3F and S3G). On the other hand, LPS did not induce phosphorylation of RAGE and its interaction with the adaptor proteins (Figures 3A and S3F) but activated the downstream molecules from TIRAP since TLR4 shares the adaptor protein with RAGE (Figures 3B and S3G). Endogenous PKCζ was activated by RAGE ligands and the activation was canceled by down regulation of RAGE using siRNA in HUVECs (Figure S3H).

It is well known that AGE induces IL-6 in endothelial cells through binding to RAGE [22]. Application of AGE to HUVECs resulted in induction of IL-6 mRNA, which was abrogated by pretreatment of the cells with inhibitory peptides for TIRAP and MyD88 (Figure 3C). When AGE was applied to a co-culture of HUVECs and human arterial sooth muscle cells (HASMCs), AGE induced IL-6 selectively in HUVECs (Figure 3D). HASMCs did not express RAGE at any appreciable levels (Figure S3A). A wider screening using an antibody array showed that angiogenin, BDNF, EGF, IL-1α, IL-1β, MIP3α, PDGF-BB, and RANTES in addition to IL-6 were induced by AGE in HUVECs and that the induction was abrogated by an inhibitor peptide for MyD88 (Figure 3E). Another well-known effect of AGE on endothelial cells is induction of apoptosis [23]. Application of AGE to HUVECs resulted in increase in annexin V-positive cell rate, which was abrogated by inhibitor peptides for TIRAP and MyD88 and by siRNA for PKCζ (Figure S3I). These results indicate that the signal transduction pathway we found in the present study, i.e., phosphorylation of RAGE by PKCζ and function of TIRAP and MyD88 as adaptor proteins, endogenously works in HUVECs, which are one of the most critical target cells of RAGE activation in physiological and pathological contexts.

### Effect of differential stimulation of RAGE and TLR4

Since TIRAP and MyD88 function as adaptor proteins for TLR2/4, we next examined the mode of signal transduction when RAGE and TLR4 were differentially stimulated. AGE and LPS pre-incubated with LBP for efficient transfer of LPS to the receptor [24] were used to selectively stimulate RAGE and TLR4, respectively, and a mixture of LPS and HMGB1 [25] or S100A8 and S100A9 (S100A8/A9) [17] were used to simultaneously stimulate both receptors. Since CD14 and MD2 have been shown to be involved in activation of TLR4 by ligands [26], we used HEK293 cells constitutively expressing both genes.

**Figure 4:**
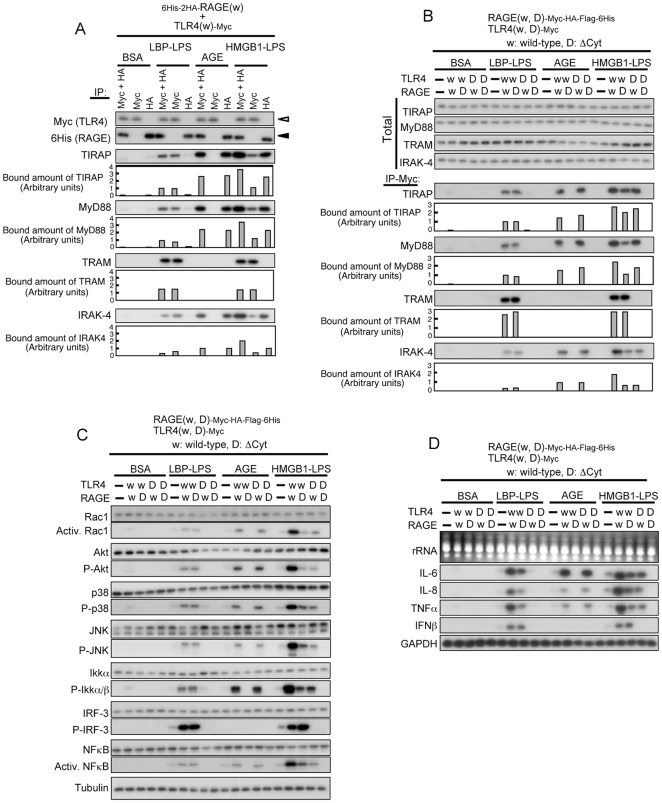
Behavior of adaptor proteins and signal transduction upon differential stimulation of RAGE and TLR4. (A) Co-precipitation of endogenous adaptor proteins with full-length 6His-2HA-RAGE(wt) and TLR4(wt)-Myc transfected into 293-hMD2-CD14 cells. The cells were treated with control BSA (100 µg/ml), LBP-LPS (100 ng/ml each, preincubated for 2 h), AGE (100 µg/ml), and HMGB1-LPS (100 ng/ml each, preincubated for 2 h) for 30 min. Cell extracts were pulled down with anti-Myc + anti-HA, anti-Myc, or anti-HA agarose and immunoblotted with indicated antibodies. Open triangle, TLR4; closed triangle, RAGE. The results were confirmed by two independent and two related experiments. (B) Co-precipitation of endogenous adaptor proteins with RAGE-Myc-HA-Flag-6His and TLR4-Myc (w, wild-type; D, cytoplasmic domain-deleted variant). Experiments were performed under conditions similar to those described in (A). The results were confirmed by two independent and two related experiments. (C) Downstream signal transduction from RAGE and TLR4. Experiments were performed under conditions similar to those described in (B). The results were confirmed by two independent and two related experiments. (D) Induction of cytokines by differential stimulation of RAGE and TLR4 as assayed by Northern blot analysis. Experiments were performed under conditions similar to those described in (B) except for treatment time (12 h). The results were confirmed by an independent and four related experiments.

Immunoprecipitation using antibodies against respective tags of TLR4 and RAGE followed by Western blot analysis showed that RAGE was co-precipitated with TIRAP, MyD88 and IRAK-4, while TLR4 was co-precipitated with TRAM in addition to the three proteins only when the receptors were activated by the ligands (Figure 4A and S5B). In accordance with the binding mode of adaptor proteins to respective receptors, downstream signaling molecules were activated (Figure S4A and S5A). Mutations in a protein-protein interaction domain, Toll/IL-1R intracellular domain (TIR), of RAGE and TLR4 (Figures S4B, S4C, and S5C) resulted in abrogation of binding to the adaptor proteins and downstream signal transduction (Figures 4B, 4C, S5C, and S5D). Activation of Rac1 and NFκB and phosphorylation of Akt, p38, JNK, and IKKα/β were observed when either RAGE or TLR4 was stimulated (Figures 4C, S4A, S4D, S5A, and S5D). Phosphorylation of IRF-3 was enhanced by stimulation of TLR4 but not by stimulation of RAGE (Figures 4C, S4A, S4D, S5A, and S5D). This is conceivable because IRF-3 is a downstream effector of TRAM and all other signaling molecules examined are downstream to TIRAP/MyD88 [27], [28]. Addition of polymixin B blocked effect of LPS but not that of AGE (Figure S4D). IL-6, IL-8 and TNFα were induced by a ligand that can bind to either RAGE or TLR4, but interferon β (IFNβ) was induced only by a TLR-ligand, HMGB1/LPS (Figure 4D). It is notable that the extent of activation of the signaling molecules and induction level of the target genes were higher when both receptors were simultaneously stimulated than that observed with stimulation of an individual receptor. These results indicate that activation of TIRAP/MyD88 triggers similar downstream signaling pathways irrespective of their binding partners.

## Discussion

**Figure 5:**
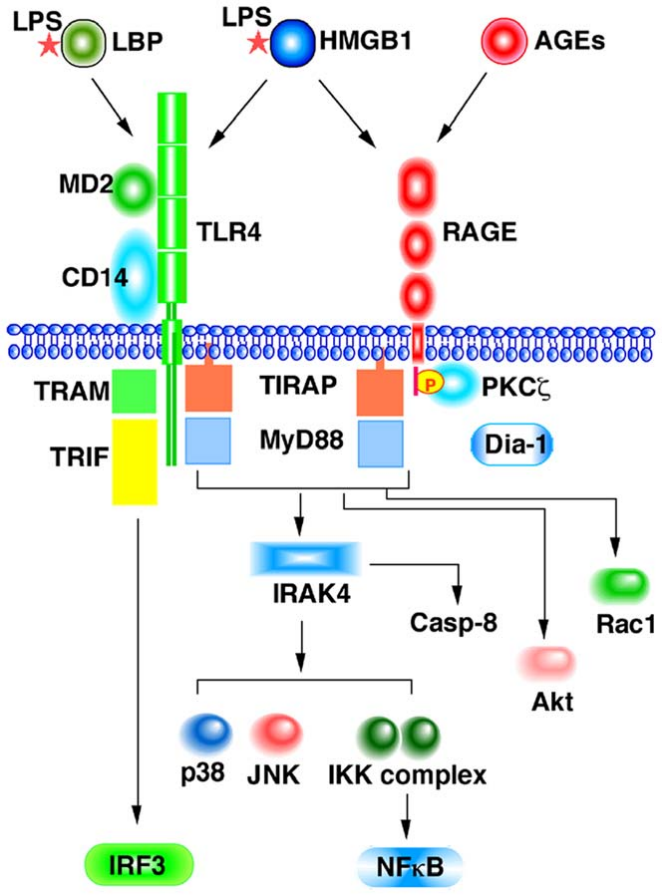
Schematic diagram of signal transduction from RAGE and TLR4. Both receptors partially share ligands and adaptor proteins. TLR4 transduces a signal to NFκB via TIRAP, MyD88 and IRAK4 and to IRF3 via TRAM and TRIF. RAGE phosphorylated by PKCζ upon ligand binding also uses TIRAP, MyD88 and IRAK4 for signal transduction to NFκB. Presence of yet unknown signaling pathway from RAGE is still possible. Simultaneous activation of both receptors resulted in enhanced activation of NFκB. The signaling machinery is also linked to Akt and caspase-8, and possibly Rac1.

The findings described above clearly demonstrate that TIRAP and MyD88 function as adaptor proteins for RAGE as well as for TLR2/4 (Figure 5). The recruitment of the adaptor proteins induced further recruitment of IRAK4, which in turn activated the downstream effecter kinases such as Akt, p38, JNK and IKKs and ultimately led to the induction or inflammatory cytokines, IL-6, IL-8, and TNFα through activation of NFκB. On the other hand, IFNβ was not induced by RAGE ligands. This result may be due to the lack of interaction between the cytoplasmic domain of RAGE and TRAM (Figure 2A). We previously showed that S100A11 could activate Akt via RAGE in keratinocytes [15]. This is probably mediated by MyD88, since MyD88 is known to directly interact with and activate the downstream effector kinase Akt [28].

Ligand-induced phosphorylation of RAGE at Ser391 is a prerequisite for efficient recruitment of the adaptor proteins. The phosphorylation was mediated by the PKCζ (Figure 1) through binding with the cytoplasmic domain of RAGE (Figure S1G). Kinase activity of PKCζ was also remarkably upregulated by stimulation of HEK293 cells with diverse RAGE ligands and of primary normal human endothelial cells (HUVECs) with AGE. AGEs are non-enzymatical adducts of proteins formed in pro-oxidant and hyperglycemic environments [2], [3], [23]. Treatment of the endothelial cells with AGE induced apoptosis (Figure S3I) [23], which was accompanied by activation of caspase-8 (Figure S3C). Notably, this effect was almost completely inhibited by siRNA for PKCζ and the inhibitor peptides for TIRAP and MyD88 (Figure S3I), indicating the critical role of these proteins in RAGE-mediated apoptotic cell death. Hence, diabetes-associated vascular damages through AGE-RAGE interaction are probably mediated by TIRAP and MyD88. It remains to be clarified how ligand-activated RAGE led to activation of PKCζ PKCζ requires activated Ras, a small GTPase, for its own kinase activity [29]. Lander et al. [9] reported that AGE-albumin activated p21 (ras) in rat pulmonary artery smooth muscle cells that express RAGE.

Hudson et al. [18] showed by immunoprecipitation that the RAGE cytoplasmic domain interacts with the FH1 domain of Dia-1 and that down-regulation of Dia-1 blocks RAGE-mediated activation of Rac-1 and Cdc42 and RAGE ligand-stimulated cellular migration. We could not detect endogenous Dia-1 protein in immunoprecipitates prepared from HEK293 cells using an antibody against tagged RAGE under the conditions in which endogenous TIRAP and MyD88 were co-precipitated with overexpressed RAGE. In accordance with the results by Hudson et al., however, overexpressed FH1 domain of Dia-1 was co-precipitated with the cytoplasmic domain of RAGE (data not shown). In addition, our preliminary data suggest that TIRAP and the FH1 domain of Dia-1 compete for binding to the cytoplasmic domain of RAGE. Precise interaction among the three proteins remains to be elucidated.

Binding of TIRAP but not MyD88 to the cytoplasmic domain of RAGE was confirmed to be direct by an *in vitro* protein interaction assay using recombinant proteins (data not shown). Phosphorylation of RAGE at Ser391 greatly enhanced affinity to TIRAP (Figure 2), indicating that more negative charge at the residue 391 is important for efficient binding with TIRAP. When the residue is phosphorylated, Q(390)SEE becomes equivalent to QEEE. Intriguingly, the motif of Q followed by 3 successive negatively charged residues is found at another site of RAGE (QEEE stating at 379) and in the cytoplasmic domain of TLR4 (QDED starting at 683) and in TLR2 (QELE starting at 661) but not of TLR1 and TLR3. The motif might play a role in interaction of intracellular domains of receptors and TIRAP.

Sharing of the adaptor proteins between RAGE and TLR2/4 is unexpected but not inconceivable. RAGE and TLR2/4 are categorized as pattern-recognizing receptors, and they use NFκB as a major intracellular signaling molecule and are involved in diverse cellular functions, inflammation in particular. Lin indicated the possibility that a TIR-containing protein may function as an adaptor protein for RAGE [20]. TLR4 has a signal transduction pathway of TRAM-TRIF in addition to TIRAP-MyD88 [30], [31]. It is not yet clear whether Dia-1 is another arm for RAGE. Both RAGE and TLR2/4 have common and distinct ligands. Our findings indicate that RAGE and TLR2/4 partly share an intracellular signaling pathway.

It is intriguing that different RAGE ligands often show different effects in given types of cells. One reason may come from simultaneous binding of RAGE ligands to TLR2/4 as described above. A complex of S100A8 and S00A9 (S100A8/A9) also binds both RAGE and TLR4 [5], [16], [32], [33]. S100A8/A9 induced phosphorylation of RAGE (Figures S1C and S1H) and led to activation of diverse signal effectors such as Rac1, Akt, p38, JNK, IKK, and NFκB (Figure S5A and S5D). On the other hand, recruitment of the adaptor proteins triggered by S100A8/A9 was limited (Figure S5B and S5C). In addition, activation of p38, JNK, IKK, and NFκB was not completely inhibited by co-expression of RAGE- and TLR4-dominant negative forms, contrary to the complete inhibition observed when treated with HMGB1-LPS (Figure S5D). This may be explained by presence of other unidentified receptor(s) for S100A8/A9 in addition to RAGE and TLR4.

Apparently, the system involving ligands-receptors-adaptor proteins for RAGE and TLR2/4 is very complicated. Further extensive studies are needed to clarify the functional interaction of proteins in the system and biological outcomes of such interactions. Studies in this direction will ultimately lead to better understanding of and development of therapeutic measures for many serious human diseases including diabetes mellitus and cancer [2]–[36].

## Materials and Methods

### Cells and Chemicals

Cell lines of HEK293, NIH3T3 (ATCC, Manassas, VA) and 293-hMD2-CD14 stably expressing human MD2 and CD14 genes (InvivoGen, San Diego, CA) and primary culture of lung fibroblasts prepared from MyD88-/- mice (Oriental Yeast, Tokyo, Japan) were cultivated in D/F medium (Invitrogen, Carlsbad, CA) supplemented with 10% FBS. HUVECs (KURABO, Osaka, Japan) were grown in Humedia-EG (KURABO) medium containing 2% FBS, FGF-B (5 ng/ml), EGF (10 ng/ml), hydrocortisone (1 µg/ml), and heparin (10 µg/ml). HASMCs (KURABO) were grown in Humedia-SG2 (KURABO) medium containing 5% FBS, FGF-B (2 ng/ml), EGF (0.5 ng/ml), and insulin (5.0 µg/ml). Cells at 3∼6 passages were used. When necessary, cells were labeled with ^32^P- orthophosphate (ICN, Costa Mesa, CA) at a dose of 100 µCi/ml for 4 h.

Sources of the materials are as follows: Lipopolysaccharide from *E. coli* 0111:B4 (LPS, Sigma-Aldrich), lipoteichoic acid from *Staphylococcus aureus* (LTA-SA), synthetic bacterial lipoprotein (Pam3CSK4), peptidoglycan from *E. coli* 0111:B4 (PGN-EC) and lipomannan from *Mycobacterium smegmatis* (LM-MS) (InvivoGen), polymixin B sulphate (PMB; Indofine Chemical Company, Hillsborough, NJ), recombinant human TLR2 extracellular domain (rexTLR2), TLR4 extracellular domain/MD-2 (rexTLR4/MD-2), LPS-binding protein (LBP) and HMGB1 (R&D Systems), AGE (made of BSA; Calbiochem, La Jolla, CA), cell-permeable peptide set comprising peptides that inhibit human TIRAP and MyD88 functions (IMGENEX, San Diego, CA), active types of recombinant MEK1, JNK1, p38α, Akt1, PKCα, PKCε, PKCδ, PKCζ, and PKCµ (MILLIPORE, Gemini Crescent Dundee, UK), recombinant IRAK4 (BPS Bioscience, San Diego, CA), and annexin V-FITC (MBL). A TLR2 ligand mixture of LTA-SA, Pam3CSK4, PGN-EC and LM-MS at 10 ng/ml each was used to activate TLR2 (TLR2Ls). A mixture of 10 µg/ml PMB, 5 µg/ml rexTLR2 and 5 µg/ml rexTLR4 was used to block activation of TLR2 and TLR4 by ligands.

### Western Blot Analysis and Immunoprecipitation

Western blot analysis was performed under conventional conditions. Antibodies used were as follows: mouse anti-HA tag (clone 6E2), mouse anti-Myc tag (clone 9B11), anti-human IRF-3; rabbit anti-human RAGE (Santa Cruz Biotechnology, Santa Cruz, CA), rabbit anti-human phospho-IRF-3 (Ser396), rabbit anti-human p38, rabbit anti-human phospho-p38 (Thr180/Tyr182), rabbit anti-human SAPK/JNK, mouse anti-human phospho-SAPK/JNK (Thr183/Tyr185), rabbit anti-human Akt, rabbit anti-human phospho-Akt (Ser473), rabbit anti-human IKKα, rabbit anti-human IKKα/β (Ser176/180), rabbit anti-human IRAK4, rabbit anti-human NFκB (p65), and mouse anti-human cleaved caspase-8 (Asp384) antibodies (Cell Signaling Technology, Beverly, MA); rabbit anti-His tag and rabbit anti-human MyD88 antibodies (MBL, Nagoya, Japan); rabbit anti-human PKCα, rabbit anti-human PKCε, rabbit anti-human PKCζ, and rabbit anti-human PKCµ antibodies (Santa Cruz Biotechnology); rabbit anti-human DIAPH1 (Dia-1), rabbit anti-human TIRAP, and mouse anti-TRAM antibodies (Abcam, Cambridge, MA); and mouse anti-human tubulin antibody (Sigma-Aldrich). Rabbit anti-human RAGE antibody was biotinylated using a Biotin Labeling Kit-SH (Dojindo Molecular Technologies, Rockville, MD) to recover antibody-free RAGE after immunoprecipitation using streptavidin-agarose (Figures. 2D, 3A, S3E and S3F). The second antibody was horseradish-peroxidase-conjugated anti-mouse or anti-rabbit IgG antibody (Cell Signaling Technology). Positive signals were detected by a chemiluminescence system (ECL plus, GE Healthcare Bio-Sciences, Piscataway, NJ). Rabbit anti-human IL-6 antibody (Abcam) was used for immunostaining.

Monoclonal Anti-HA (clone HA-7) tag-agarose (Sigma-Aldrich), monoclonal Anti-His tag (clone 2D8) and monoclonal anti-Myc tag (clone 1G4) agaroses (MBL), and streptavidin agarose (Invitrogen) were used for the co-immunoprecipitation experiments.

### Northern Blot Analysis

Ten micrograms of total RNA isolated by the acid guanidinium thiocyanate/phenol-chloroform method was electrophoresed in a 1% agarose gel and transferred to a Nytran Plus nylon membrane (GE Healthcare Bio-Sciences). Entire coding regions of human IL-6, IL-8, TNFα, IFNβ, and GAPDH genes were used as probes for Northern blot analysis.

### Plasmid Constructs

Human cDNAs, S100A12, HMGB1, and cytoplasmic domain of RAGE (RAGE(Cyt), 364-404 aa) were cloned into pGEX vector series (GE Healthcare Bio-Sciences), i.e., S100A12 into pGEX-6P3, HMGB1 and RAGE(Cyt) into pGEX-6P1.

We made a mammalian expression vector by modifying the pIDT-SMART cloning vector (Integrated Device Technology, San Jose, CA) using CMV promoter-intron (CMVi) and a part of the HTLV type 1 LTR (RU5') to express even shorter cargo cDNAs at very high levels. Various combinations of tags at either the N-terminal (6His-2HA) or C-terminal (Myc-HA-Flag-6His) side of RAGE and its variant cDNAs were prepared. Since RAGE is a single-pass type I membrane protein, the tag sequence was put between the N-terminal region (1–31 aa) including the signal sequence (1–23 aa) and the remaining part of RAGE cDNA when constructing N-terminally tagged vectors (Figure. S1F). PKC isoforms were tagged with C-terminal HA, and TLR adaptor proteins, TIRAP, MyD88 and TRAM, were tagged with N-terminal Myc and C-terminal HA.

### Recombinant Protein Preparation

Dimerized human S100A11 was prepared as described previously [15]. Purification of the bacterially expressed GST-fusion proteins was performed under conventional conditions. GST was cleaved with PreScission protease (GE Healthcare Bio-Sciences) when necessary. Endotoxin (LPS and β-glucans) contaminated in the purified recombinant proteins were determined by a Limulus Amebocyte Lysate (LAL) assay using a Limulus test kit (Seikagaku Corporation, Tokyo, Japan). Detected amounts were as follows: GST, 0.013 EU/µg; HMGB1, 0.017 EU/µg; S100A11, 0.007 EU/µg; S100A12, 0.008 EU/µg; AGE-BSA (Calbiochem), 0.001 EU/µg; and HMGB1 (R&D), 0.001 EU/µg.

### *In vitro* Kinase Assay

*In vitro* kinase reaction was performed using active type of recombinant kinases (50 ng) and purified GST-RAGE(Cyt) (1 µg) or HEK293-derived RAGE(Cyt) purified by anti-6His tag antibody agarose (MBL) in the presence of [γ-^32^P] ATP (HAS, Budapest, Hungary). To confirm activation of PKCζ in cells, biotinylated-PKCζ–specific substrate (Santa Cruz Biotechnology) was incubated with cell extracts, and then the substrate peptide was trapped on a streptavidin-coated plate (BD Biosciences Bedford, MA) and radioactivity was determined.

### Assay for Activated NFκB and Rac1

Pull-down assay of NFκB transcription factor bound to the NFκB-responsive element (NFκB RE) was performed under conditions reported previously [15]. A biotinylated 22-bp NFκB RE oligo DNA was prepared using a forward primer (5′-biotin-AGTTGA**GGGGACTTTCCC**AGGC -3′) and a reverse primer (3′-TCAACT**CCCCTGAAAGGG**TCCG-biotin-5′), incubated with whole cell extracts, and pulled down using a streptavidine agarose (Invitrogen). Bound NFκB (p65) was determined by Western blot analysis. GTP-bound form of Rac1 was determined using a Rac1 Activation Assay Kit (MILLIPORE).

### RNA Interference

Validated siRNAs for human PKCα (ID No. s11092) and for human PKCζ (ID No. s11129) and control siRNA (Silencer Negative control 2 siRNA) were purchased from Ambion (Austin, TX). Human PKCε (siGENOME SMART pool M-004653-02-0005), human PKCµ (siGENOME SMART pool M-005028-02-0005), human RAGE (siGENOME SMART pool M-003625-02-0005) and mouse TIRAP (siGENOME SMART pool M-049112-00-0005) siRNAs were purchased from Thermo Scientific Dharmacon (Lafayette, CO). siRNAs were transfected using Lipofectamin RNAiMAX reagent (Invitrogen).

## Supporting Information

Figure S1 **Phosphorylation of RAGE and identification of a responsible kinase.** (A and B) Phosphorylation of full-length RAGE (wt) but not cytoplasmic domain-deleted variant (ΔCyt). HEK293 cells were transfected with plasmid constructs of RAGE-Myc-HA-Flag-6His (wt, closed arrowhead; ΔCyt, open arrowhead), and the cells were then treated with 0∼100 ng/ml of recombinant S100A11 protein for 1 h (A: The results were confirmed by an independent and four related experiments.) or with 10 ng/ml of the protein for indicated periods (B: The results were confirmed by an independent and four related experiments.). [^32^P]Phosphate-labeled cell extracts were pulled-down with anti-HA agarose and immunoblotted or analyzed by autoradiography. (C) Phosphorylation of full-length RAGE (wt) by S100A8/A9. Experiments were performed under the conditions similar to those described in Figure 1A. HEK293 cells were exposed to 10 ng/ml of GST or a mixture of S100A8 and S100A9 recombinant proteins (S100A8/A9; 10 ng/ml each) for 30 min. +PMB indicates that the recombinant proteins were preincubated with polymixin B (10 µg/ml) in order to abrogate possible effect of LPS. The results were confirmed by three independent experiments. (D) Phosphorylation of RAGE (Cyt) by recombinant kinases (constitutively active form) *in vitro*. : The results were confirmed by an independent and three related experiments. (E) Confirmation of the specificity of biotinylated PKCζ substrate. Recombinant kinases (constitutively active form) were incubated with the PKCζ-substrate in the presence of [γ-^32^P]-ATP and the substrate was trapped on a streptavidin-coated 96-well plate. The experiment was performed in quadruplicates. (F) Dose-dependent activation of PKCζ by RAGE ligands in RAGE-transfected HEK293 cells. Experimental conditions were similar to those for Fig. 1E. Standard deviations were negligible on this scale. The results were representative of two independent experiments. (G) Effect of RAGE phosphorylation on co-precipitation with PKC isoforms. Upper panel: A diagram of N-terminally 6His-2HA-tagged full-length RAGE. SS, signal sequence; Ext D, extracellular domain; TMD, transmembrane domain; Cyt D, cytoplasmic domain. Lower panel: Plasmid constructs of 6His-2HA-RAGE(Cyt) variants [SSST (wild-type), ASST (Ser391 to Ala; non-phosphorylatable), and ESST (Ser391 to Glu; phosphorylation mimic)] were co-transfected with PKC isoforms (PKCs-HA) into HEK293 cells. Amounts of PKCs (using upper part of the gel) and RAGE (using lower part of the gel) were determined with anti-HA antibody with or without immunoprecipitation with anti-6His antibody. The results are representative of three independent experiments. (H) Down-regulation of endogenous PKCζ by siRNA abrogated S100A8/A9 (30 min)-induced phosphorylation of co-transfected RAGE-Myc-HA-Flag-6His in HEK293 cells. The results were confirmed by two independent and three related experiments.
(TIF)

Figure S2 **Identification of adaptor proteins for RAGE.** (A) Co-precipitation of TIRAP with RAGE(Cyt). Plasmid constructs of 6His-2HA-RAGE(Cyt) and Myc-adaptors (MyD88, TIRAP, or TRAM)-HA were co-transfected into HEK293 cells and the cell extracts were analyzed by immunoblotting for their expression (upper panel) and for co-precipitation (lower panel) after pulling down with anti-6His agarose. The results were confirmed by two independent and six related experiments. (B) Effect of RAGE phosphorylation status on binding with adaptor proteins. Plasmid constructs of 6His-2HA-RAGE(Cyt) variants (see the legend for Fig. S1F) were co-transfected with Myc-MyD88-HA and Myc-TIRAP-HA into HEK293 cells and analyzed in a way similar to that described in (A). Closed arrowhead, MyD88; open arrowhead, TIRAP. The results were confirmed by an independent and four related experiments. (C) Mutation in the TIR domain of TIRAP (^118^LQLRDATPGGAIV^131^S replaced with SQSSSATGGGASGS) and MyD88 (^196^RDVLPG^202^T replaced with SSGLGGT) abrogated their binding to 6His-2HA-RAGE(Cyt) in co-transfected HEK293 cells. W, wild-type; M, mutated. Closed arrowhead, MyD88; open arrowhead, TIRAP. The results were confirmed by three independent and five related experiments. (D) No effect of AGE on expression levels of MyD88 and TIRAP in TIRAP-down regulated NIH3T3 cells and MyD88-/- fibroblasts, respectively. Mouse TIRAP siRNA was applied at 10 nM for 48 h. The results were confirmed by an independent and a related experiments.
(TIF)

Figure S3 **Activation of endogenous RAGE and its biological consequence in HUVECs.** (A) Expression levels of endogenous RAGE in normal human keratinocytes (NHK), HUVECs, and HASMCs determined by Western blot analysis. The results were representative of four independent experiments. (B and C) Intracellular signal transduction triggered in HUVECs treated with AGE at different doses (B) and for different periods (C). The results were confirmed by an independent and two related experiments. (D) Inhibition of AGE-induced nuclear translocation of NFκB by cell-permeable inhibitory peptides for MyD88 and TIRAP (100 µM) in HUVECs. The results are representative of two independent experiments. (E) Immunoprecipitation with biotinylated anti-RAGE antibody enabled to recover RAGE protein without contamination with the first antibody. The results were confirmed by two independent and three related experiments. (F and G) Effect of interference with functions of downstream molecules on signaling triggered from activated endogenous RAGE and TLR. Cell-permeable inhibitory peptides (100 µM) for TIRAP and MyD88 or siRNAs (10 nM) for PKCζ were applied 12 h or 48 h prior to stimulation with AGE (100 µg/ml for 1 h) or LPS (100 ng/ml for 1 h). BSA was used as a control. The results were confirmed by an independent and two related experiments (F) or by an independent and three related experiments (G). (H) Activation of PKCζ on stimulation of endogenous RAGE with various ligands. Experiments were performed under the conditions similar to those described in the legend for Figs. 3A, 3B and 1E. The results were confirmed by an independent experiment. (I) Induction of apoptosis by AGE in HUVECs was abrogated by inhibiting TIRAP, MyD88 or PKCζ. HUVECs were treated with 100 µg/ml AGE for 48 h. Cell-permeable inhibitory peptides (100 µM) were added at 12 h prior to and twice during the treatment with AGE. siRNA for PKCζ was applied 24 h prior to application of AGE. Experiments were performed under serum-free conditions but in the presence of supplements. Apoptosis was determined by staining with annexin V-FITC. The experiment was performed in triplicates. The results were confirmed by an independent experiment.
(TIF)

Figure S4 **Analysis of differential stimulation of RAGE and TLR4.** (A) Intracellular signal transduction triggered by ligands stimulating overexpressed 6His-2HA-RAGE and TLR4-Myc in 293-hMD2-CD14 cells examined under conditions described in Fig. 4A. Open triangle, TLR4; closed triangle, RAGE. The results were confirmed by two independent and three related experiments. (B) Expression of RAGE-Myc-HA-Flag-6His and TLR4-Myc (w, wild-type; D, cytoplasmic domain-deleted) in 293-hMD2-CD14 cells determined using anti-Myc antibody. The results were confirmed by two independent and three related experiments. (C) Expression of RAGE-Myc-HA-Flag-6His and TLR4-Myc (w, wild-type; D, cytoplasmic domain-deleted) in 293-hMD2-CD14 cells treated with AGE (100 µg/ml) and other agents (100 ng/ml), as assayed by immunoprecipitation followed by Western blotting using anti-Myc antibody. The results were confirmed by two independent and three related experiments. (D) Abrogation of the effect of LPS but not of AGE by polymixin B (PMB). Experiments were performed under conditions similar to Figs. 4B and 4C. Open triangle, TLR4; closed triangle, RAGE. The results were confirmed by an independent and three related experiments.
(TIF)

Figure S5 **Behavior of adaptor proteins and signal transduction upon S100A8/A9 stimulation.** (A) Intracellular signal transduction triggered by ligands in 293-hMD2-CD14 cells overexpressed 6His-2HA-RAGE and TLR4-Myc under same conditions described in Fig. S4A. The transfected cells were treated with control BSA (100 µg/ml), LBP-LPS (100 ng/ml each, preincubated for 2 h), AGE (100 µg/ml), HMGB1-LPS (100 ng/ml each, preincubated for 2 h), and S100A8/A9 (100 ng/ml each) for 30 min. Open triangle, TLR4; closed triangle, RAGE. The results were confirmed by two independent and three related experiments. (B) Co-precipitation of endogenous adaptor proteins with full-length 6His-2HA-RAGE (wt) and TLR4 (wt)-Myc transfected into 293-hMD2-CD14 cells were performed under the conditions similar to those described in Fig. 4A. Cell extracts were pulled down with anti-Myc + anti-HA, anti-Myc, or anti-HA agarose and immunoblotted with indicated antibodies. Open triangle, TLR4; closed triangle, RAGE as corresponding to (A). The results were confirmed by two independent and two related experiments. (C) Co-precipitation of endogenous adaptor proteins with RAGE-Myc-HA-Flag-6His and TLR4-Myc (w, wild-type; D, cytoplasmic domain-deleted variant) in 293-hMD2-CD14 cells treated with the same series of agents indicated in (A) and (B). Experiments were performed by immunoprecipitation followed by Western blotting using anti-Myc antibody as described in Figs.S4C and 4B. The results were confirmed by two independent and two related experiments. (D) Downstream signal transduction from RAGE and TLR4 stimulated with S100A8/A9. Experiments were performed under the conditions similar to those described in Fig. 4C. The results were confirmed by two independent and three related experiments.
(TIF)

## References

[1] Neeper M, Schmidt AM, Brett J, Yan SD, Wang F (1992). Cloning and expression of a cell surface receptor for advanced glycosylation end products of proteins.. J Biol Chem.

[2] Schmidt AM, Hori O, Cao R, Yan SD, Brett J (1996). RAGE: a novel cellular receptor for advanced glycation end products.. Diabetes.

[3] Yan SF, Ramasamy R, Schmidt AM (2008). Mechanisms of disease: advanced glycation end-products and their receptor in inflammation and diabetes complications.. Nat Clin Pract Endocrinol Metab.

[4] Lin L, Park S, Lakatta EG (2009). RAGE signaling in inflammation and arterial aging.. Front Biosci.

[5] Sims GP, Rowe DC, Rietdijk ST, Herbst R, Coyle AJ (2010). HMGB1 and RAGE in inflammation and cancer.. Annu Rev Immunol.

[6] Hori O, Brett J, Slattery T, Cao R, Zhang J (1995). The receptor for advanced glycation end products (RAGE) is a cellular binding site for amphoterin. Mediation of neurite outgrowth and co-expression of rage and amphoterin in the developing nervous system.. J Biol Chem.

[7] Leclerc E, Fritz G, Vetter SW, Heizmann CW (2009). Binding of S100 proteins to RAGE: an update.. Biochim Biophys Acta.

[8] Leclerc E, Sturchler E, Vetter SW, Heizmann CW (2009). Crosstalk between calcium, amyloid beta and the receptor for advanced glycation endproducts in Alzheimer's disease.. Rev Neurosci.

[9] Lander HM, Tauras JM, Ogiste JS, Hori O, Moss RA (1997). Activation of the receptor for advanced glycation end products triggers a p21(ras)-dependent mitogen-activated protein kinase pathway regulated by oxidant stress.. J Biol Chem.

[10] Leclerc E, Fritz G, Weibel M, Heizmann CW, Galichet A (2007). S100B and S100A6 differentially modulate cell survival by interacting with distinct RAGE (receptor for advanced glycation end products) immunoglobulin domains.. J Biol Chem.

[11] Taguchi A, Blood DC, del Toro G, Canet A, Lee DC (2000). Blockade of RAGE-amphoterin signalling suppresses tumour growth and metastases.. Nature.

[12] Yeh CH, Sturgis L, Haidacher J, Zhang XN, Sherwood SJ (2001). Requirement for p38 and p44/p42 mitogen-activated protein kinases in RAGE-mediated nuclear factor-kappaB transcriptional activation and cytokine secretion.. Diabetes.

[13] Fages C, Nolo R, Huttunen HJ, Eskelinen E, Rauvala H (2000). Regulation of cell migration by amphoterin.. J Cell Sci.

[14] Gebhardt C, Riehl A, Durchdewald M, Nemeth J, Furstenberger G (2008). RAGE signaling sustains inflammation and promotes tumor development.. J Exp Med.

[15] Sakaguchi M, Sonegawa H, Murata H, Kitazoe M, Futami J (2008). S100A11, an dual mediator for growth regulation of human keratinocytes.. Mol Biol Cell.

[16] Ghavami S, Rashedi I, Dattilo BM, Eshraghi M, Chazin WJ (2008). S100A8/A9 at low concentration promotes tumor cell growth via RAGE ligation and MAP kinase-dependent pathway.. J Leukoc Biol.

[17] Nukui T, Ehama R, Sakaguchi M, Sonegawa H, Katagiri C (2008). S100A8/A9, a key mediator for positive feedback growth stimulation of normal human keratinocytes.. J Cell Biochem.

[18] Hudson BI, Kalea AZ, Del Mar Arriero M, Harja E, Boulanger E (2008). Interaction of the RAGE cytoplasmic domain with diaphanous-1 is required for ligand-stimulated cellular migration through activation of Rac1 and Cdc42.. J Biol Chem.

[19] Ishihara K, Tsutsumi K, Kawane S, Nakajima M, Kasaoka T (2003). The receptor for advanced glycation end-products (RAGE) directly binds to ERK by a D-domain-like docking site.. FEBS Lett.

[20] Lin L (2006). RAGE on the Toll Road?. Cell Mol Immunol.

[21] Ohnishi H, Tochio H, Kato Z, Orii KE, Li A (2009). Structural basis for the multiple interactions of the MyD88 TIR domain in TLR4 signaling.. Proc Natl Acad Sci U S A.

[22] Schmidt AM, Hasu M, Popov D, Zhang JH, Chen J (1994). Receptor for advanced glycation end products (AGEs) has a central role in vessel wall interactions and gene activation in response to circulating AGE proteins.. Proc Natl Acad Sci U S A.

[23] Zhou YJ, Wang JH, Zhang J (2006). Hepatocyte growth factor protects human endothelial cells against advanced glycation end products-induced apoptosis.. Biochem Biophys Res Commun.

[24] Schumann RR, Leong SR, Flaggs GW, Gray PW, Wright SD (1990). Structure and function of lipopolysaccharide binding protein.. Science.

[25] Youn JH, Oh YJ, Kim ES, Choi JE, Shin JS (2008). High mobility group box 1 protein binding to lipopolysaccharide facilitates transfer of lipopolysaccharide to CD14 and enhances lipopolysaccharide-mediated TNF-alpha production in human monocytes.. J Immunol.

[26] Akashi-Takamura S, Miyake K (2008). TLR accessory molecules.. Curr Opin Immunol.

[27] Kawai T, Akira S (2006). TLR signaling.. Cell Death Differ.

[28] van Beijnum JR, Buurman WA, Griffioen AW (2008). Convergence and amplification of toll-like receptor (TLR) and receptor for advanced glycation end products (RAGE) signaling pathways via high mobility group B1 (HMGB1).. Angiogenesis.

[29] Fedorov YV, Jones NC, Olwin BB (2002). Atypical protein kinase Cs are the Ras effectors that mediate repression of myogenic satellite cell differentiation.. Mol Cell Biol.

[30] O'Neill LA, Bowie AG (2007). The family of five: TIR-domain-containing adaptors in Toll-like receptor signalling.. Nat Rev Immunol.

[31] Gay NJ, Gangloff M (2007). Structure and function of Toll receptors and their ligands.. Annu Rev Biochem.

[32] Vogl T, Tenbrock K, Ludwig S, Leukert N, Ehrhardt C (2007). Mrp8 and Mrp14 are endogenous activators of Toll-like receptor 4, promoting lethal, endotoxin-induced shock.. Nat Med.

[33] Loser K, Vogl T, Voskort M, Lueken A, Kupas V (2010). The Toll-like receptor 4 ligands Mrp8 and Mrp14 are crucial in the development of autoreactive CD8+ T cells.. Nat Med.

[34] Jagannathan M, Hasturk H, Liang Y, Shin H, Hetzel JT (2009). TLR cross-talk specifically regulates cytokine production by B cells from chronic inflammatory disease patients.. J Immunol.

[35] Drexler SK, Foxwell BM (2010). The role of toll-like receptors in chronic inflammation.. Int J Biochem Cell Biol.

[36] Fukata M, Shang L, Santaolalla R, Sotolongo J, Pastorini C (2011). Constitutive activation of epithelial TLR4 augments inflammatory responses to mucosal injury and drives colitis-associated tumorigenesis.. Inflamm Bowel Dis.

