# Supplementary figures and images for: TIRAP, an Adaptor Protein for TLR2/4, Transduces a Signal from RAGE Phosphorylated upon Ligand Binding
Source: PLoS One. 2011 Aug 1;6(8):e23132. doi: 10.1371/journal.pone.0023132 (PMC3148248; doi:10.1371/journal.pone.0023132)

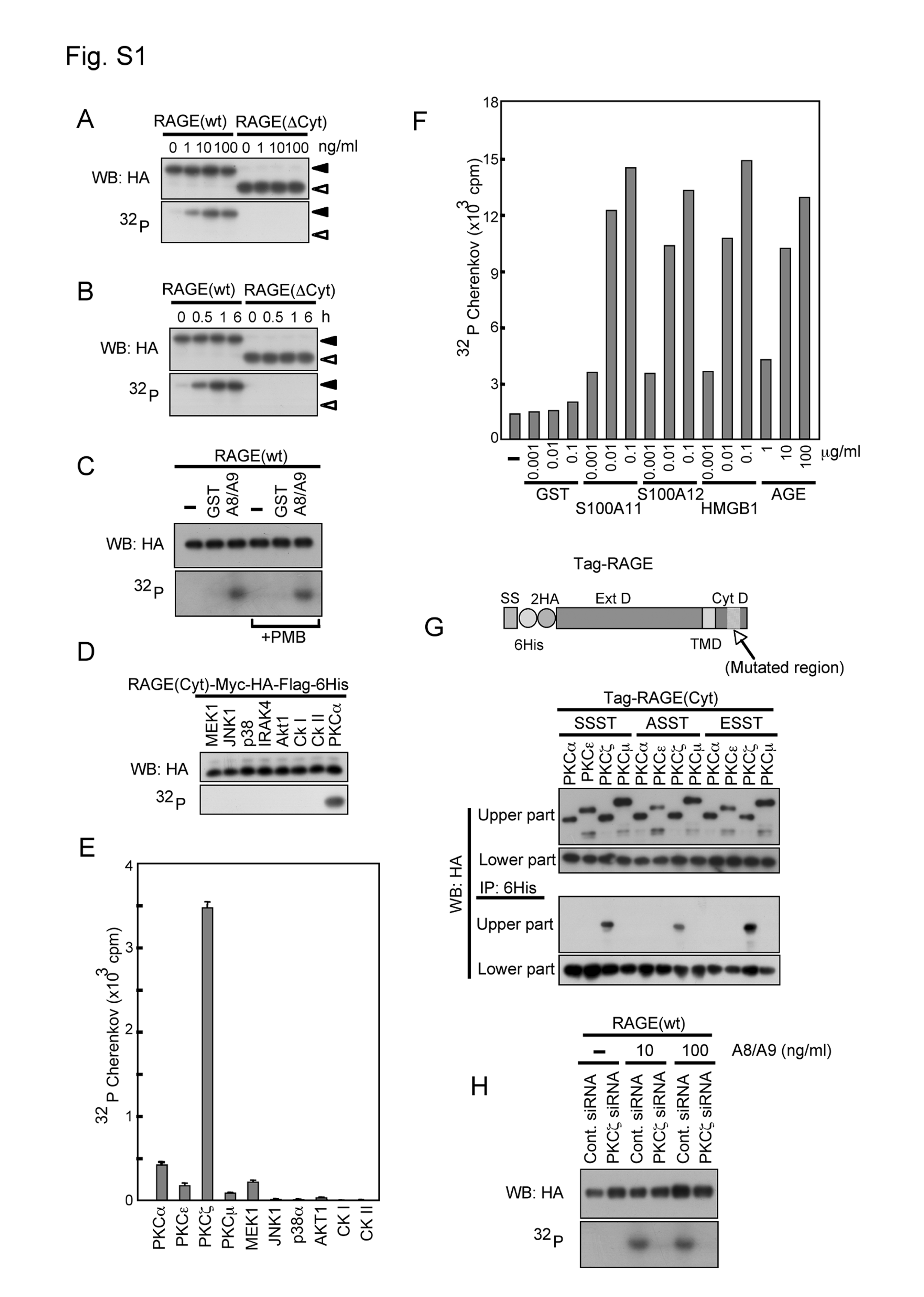

Supplement: Figure S1 — Phosphorylation of RAGE and identification of a responsible kinase. (A and B) Phosphorylation of full-length RAGE (wt) but not cytoplasmic domain-deleted variant (ΔCyt). HEK293 cells were transfected with plasmid constructs of RAGE-Myc-HA-Flag-6His (wt, closed arrowhead; ΔCyt, open arrowhead), and the cells were then treated with 0∼100 ng/ml of recombinant S100A11 protein for 1 h (A: The results were confirmed by an independent and four related experiments.) or with 10 ng/ml of the protein for indicated periods (B: The results were confirmed by an independent and four related experiments.). [32P]Phosphate-labeled cell extracts were pulled-down with anti-HA agarose and immunoblotted or analyzed by autoradiography. (C) Phosphorylation of full-length RAGE (wt) by S100A8/A9. Experiments were performed under the conditions similar to those described in Figure 1A. HEK293 cells were exposed to 10 ng/ml of GST or a mixture of S100A8 and S100A9 recombinant proteins (S100A8/A9; 10 ng/ml each) for 30 min. +PMB indicates that the recombinant proteins were preincubated with polymixin B (10 µg/ml) in order to abrogate possible effect of LPS. The results were confirmed by three independent experiments. (D) Phosphorylation of RAGE (Cyt) by recombinant kinases (constitutively active form) in vitro. : The results were confirmed by an independent and three related experiments. (E) Confirmation of the specificity of biotinylated PKCζ substrate. Recombinant kinases (constitutively active form) were incubated with the PKCζ-substrate in the presence of [γ-32P]-ATP and the substrate was trapped on a streptavidin-coated 96-well plate. The experiment was performed in quadruplicates. (F) Dose-dependent activation of PKCζ by RAGE ligands in RAGE-transfected HEK293 cells. Experimental conditions were similar to those for Fig. 1E. Standard deviations were negligible on this scale. The results were representative of two independent experiments. (G) Effect of RAGE phosphorylation on co-precipitati [file pone.0023132.s001.tif]

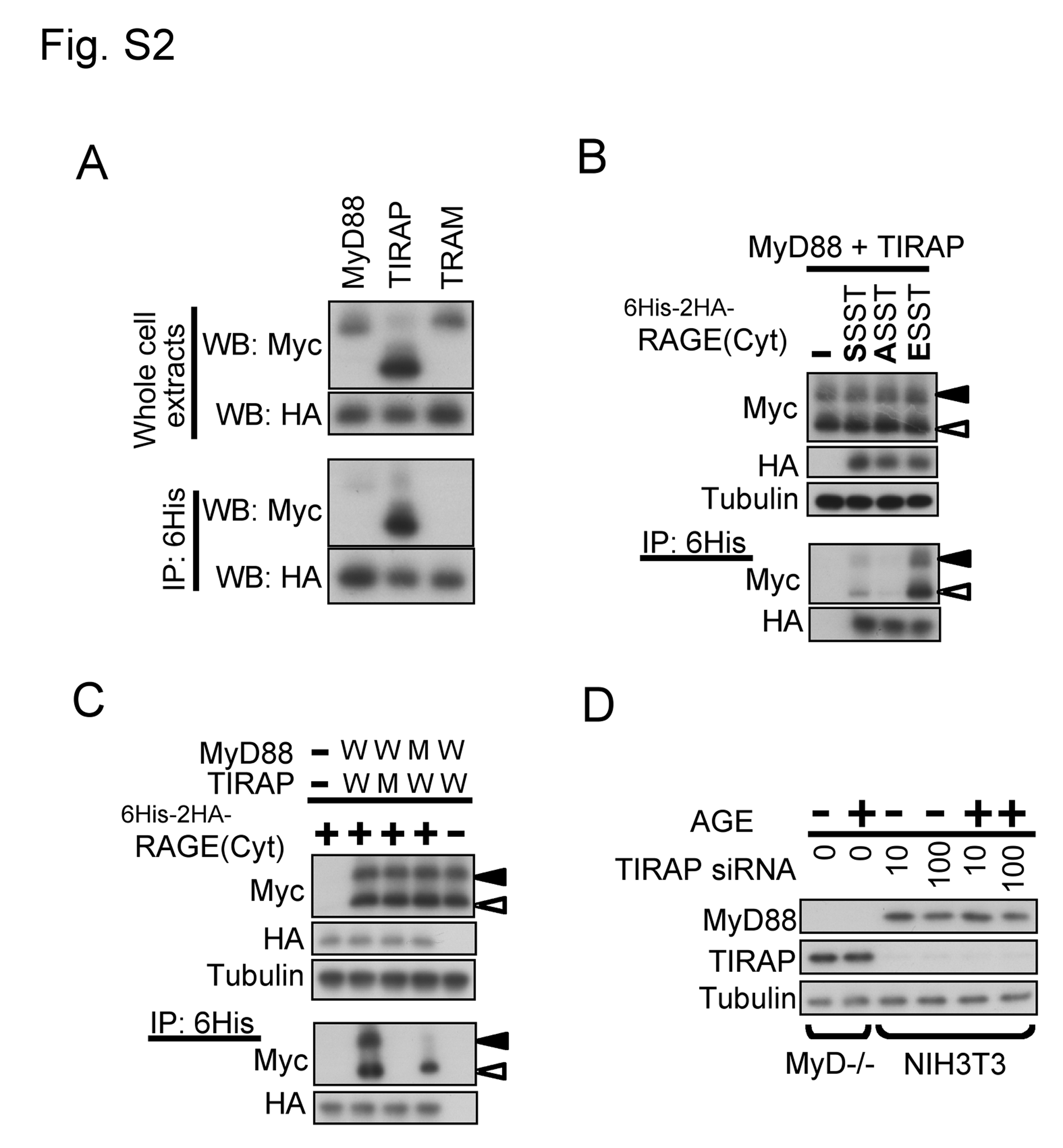

Supplement: Figure S2 — Identification of adaptor proteins for RAGE. (A) Co-precipitation of TIRAP with RAGE(Cyt). Plasmid constructs of 6His-2HA-RAGE(Cyt) and Myc-adaptors (MyD88, TIRAP, or TRAM)-HA were co-transfected into HEK293 cells and the cell extracts were analyzed by immunoblotting for their expression (upper panel) and for co-precipitation (lower panel) after pulling down with anti-6His agarose. The results were confirmed by two independent and six related experiments. (B) Effect of RAGE phosphorylation status on binding with adaptor proteins. Plasmid constructs of 6His-2HA-RAGE(Cyt) variants (see the legend for Fig. S1F) were co-transfected with Myc-MyD88-HA and Myc-TIRAP-HA into HEK293 cells and analyzed in a way similar to that described in (A). Closed arrowhead, MyD88; open arrowhead, TIRAP. The results were confirmed by an independent and four related experiments. (C) Mutation in the TIR domain of TIRAP (118LQLRDATPGGAIV131S replaced with SQSSSATGGGASGS) and MyD88 (196RDVLPG202T replaced with SSGLGGT) abrogated their binding to 6His-2HA-RAGE(Cyt) in co-transfected HEK293 cells. W, wild-type; M, mutated. Closed arrowhead, MyD88; open arrowhead, TIRAP. The results were confirmed by three independent and five related experiments. (D) No effect of AGE on expression levels of MyD88 and TIRAP in TIRAP-down regulated NIH3T3 cells and MyD88-/- fibroblasts, respectively. Mouse TIRAP siRNA was applied at 10 nM for 48 h. The results were confirmed by an independent and a related experiments. (TIF) [file pone.0023132.s002.tif]

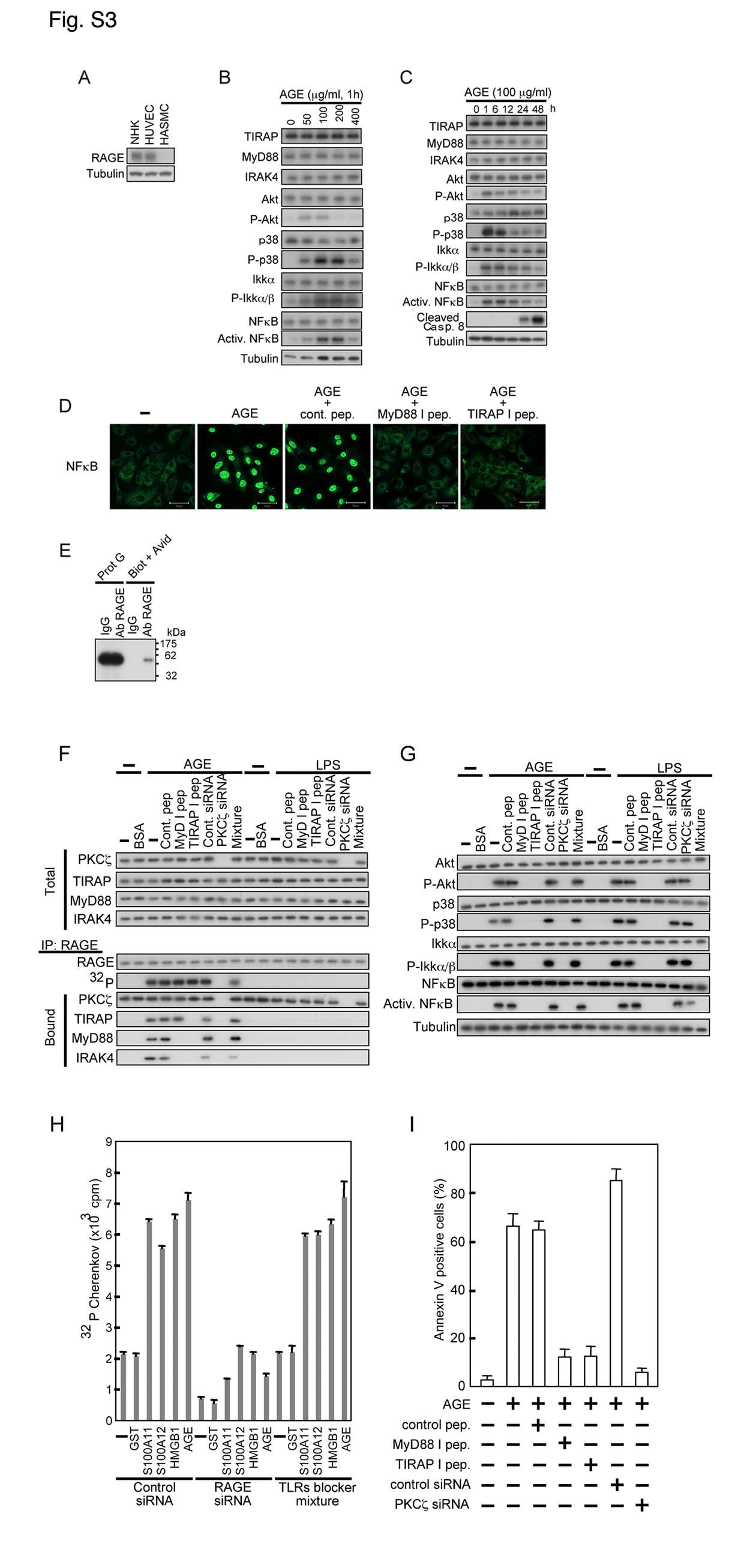

Supplement: Figure S3 — Activation of endogenous RAGE and its biological consequence in HUVECs. (A) Expression levels of endogenous RAGE in normal human keratinocytes (NHK), HUVECs, and HASMCs determined by Western blot analysis. The results were representative of four independent experiments. (B and C) Intracellular signal transduction triggered in HUVECs treated with AGE at different doses (B) and for different periods (C). The results were confirmed by an independent and two related experiments. (D) Inhibition of AGE-induced nuclear translocation of NFκB by cell-permeable inhibitory peptides for MyD88 and TIRAP (100 µM) in HUVECs. The results are representative of two independent experiments. (E) Immunoprecipitation with biotinylated anti-RAGE antibody enabled to recover RAGE protein without contamination with the first antibody. The results were confirmed by two independent and three related experiments. (F and G) Effect of interference with functions of downstream molecules on signaling triggered from activated endogenous RAGE and TLR. Cell-permeable inhibitory peptides (100 µM) for TIRAP and MyD88 or siRNAs (10 nM) for PKCζ were applied 12 h or 48 h prior to stimulation with AGE (100 µg/ml for 1 h) or LPS (100 ng/ml for 1 h). BSA was used as a control. The results were confirmed by an independent and two related experiments (F) or by an independent and three related experiments (G). (H) Activation of PKCζ on stimulation of endogenous RAGE with various ligands. Experiments were performed under the conditions similar to those described in the legend for Figs. 3A, 3B and 1E. The results were confirmed by an independent experiment. (I) Induction of apoptosis by AGE in HUVECs was abrogated by inhibiting TIRAP, MyD88 or PKCζ. HUVECs were treated with 100 µg/ml AGE for 48 h. Cell-permeable inhibitory peptides (100 µM) were added at 12 h prior to and twice during the treatment with AGE. siRNA for PKCζ was applied 24 h prior to application of AGE. Experiments were performed under serum-free c [file pone.0023132.s003.tif]

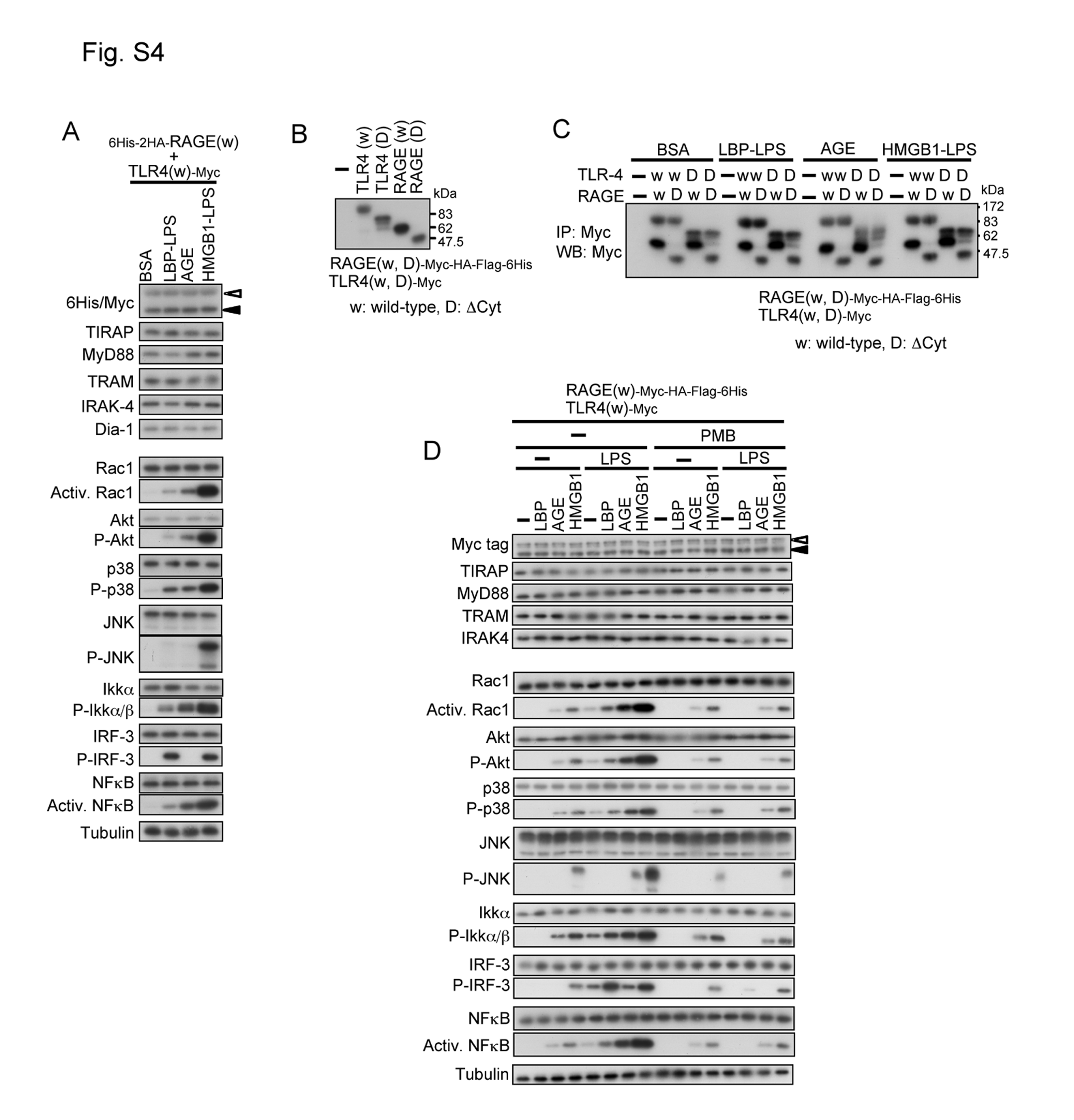

Supplement: Figure S4 — Analysis of differential stimulation of RAGE and TLR4. (A) Intracellular signal transduction triggered by ligands stimulating overexpressed 6His-2HA-RAGE and TLR4-Myc in 293-hMD2-CD14 cells examined under conditions described in Fig. 4A. Open triangle, TLR4; closed triangle, RAGE. The results were confirmed by two independent and three related experiments. (B) Expression of RAGE-Myc-HA-Flag-6His and TLR4-Myc (w, wild-type; D, cytoplasmic domain-deleted) in 293-hMD2-CD14 cells determined using anti-Myc antibody. The results were confirmed by two independent and three related experiments. (C) Expression of RAGE-Myc-HA-Flag-6His and TLR4-Myc (w, wild-type; D, cytoplasmic domain-deleted) in 293-hMD2-CD14 cells treated with AGE (100 µg/ml) and other agents (100 ng/ml), as assayed by immunoprecipitation followed by Western blotting using anti-Myc antibody. The results were confirmed by two independent and three related experiments. (D) Abrogation of the effect of LPS but not of AGE by polymixin B (PMB). Experiments were performed under conditions similar to Figs. 4B and 4C. Open triangle, TLR4; closed triangle, RAGE. The results were confirmed by an independent and three related experiments. (TIF) [file pone.0023132.s004.tif]

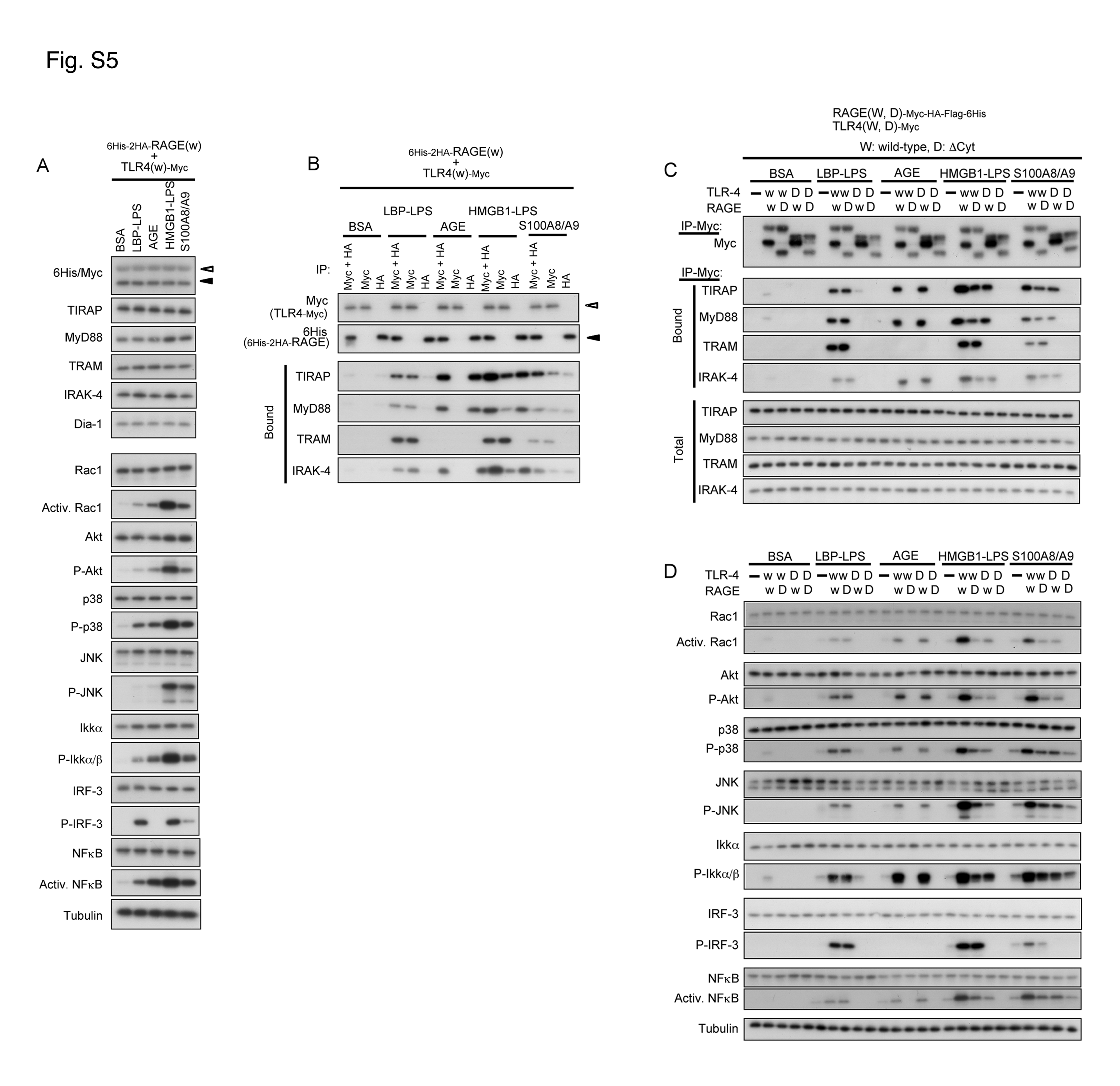

Supplement: Figure S5 — Behavior of adaptor proteins and signal transduction upon S100A8/A9 stimulation. (A) Intracellular signal transduction triggered by ligands in 293-hMD2-CD14 cells overexpressed 6His-2HA-RAGE and TLR4-Myc under same conditions described in Fig. S4A. The transfected cells were treated with control BSA (100 µg/ml), LBP-LPS (100 ng/ml each, preincubated for 2 h), AGE (100 µg/ml), HMGB1-LPS (100 ng/ml each, preincubated for 2 h), and S100A8/A9 (100 ng/ml each) for 30 min. Open triangle, TLR4; closed triangle, RAGE. The results were confirmed by two independent and three related experiments. (B) Co-precipitation of endogenous adaptor proteins with full-length 6His-2HA-RAGE (wt) and TLR4 (wt)-Myc transfected into 293-hMD2-CD14 cells were performed under the conditions similar to those described in Fig. 4A. Cell extracts were pulled down with anti-Myc + anti-HA, anti-Myc, or anti-HA agarose and immunoblotted with indicated antibodies. Open triangle, TLR4; closed triangle, RAGE as corresponding to (A). The results were confirmed by two independent and two related experiments. (C) Co-precipitation of endogenous adaptor proteins with RAGE-Myc-HA-Flag-6His and TLR4-Myc (w, wild-type; D, cytoplasmic domain-deleted variant) in 293-hMD2-CD14 cells treated with the same series of agents indicated in (A) and (B). Experiments were performed by immunoprecipitation followed by Western blotting using anti-Myc antibody as described in Figs.S4C and 4B. The results were confirmed by two independent and two related experiments. (D) Downstream signal transduction from RAGE and TLR4 stimulated with S100A8/A9. Experiments were performed under the conditions similar to those described in Fig. 4C. The results were confirmed by two independent and three related experiments. (TIF) [file pone.0023132.s005.tif]
